# Moving fast but going slow: coordination challenges for trials of COVID-19 post-exposure prophylaxis

**DOI:** 10.1186/s13063-020-04754-9

**Published:** 2020-09-29

**Authors:** Darrell H. S. Tan, Rupesh Agrawal, Ruanne V. Barnabas, Jon T. Giles, Peter Dull

**Affiliations:** 1 Division of Infectious Diseases, St. Michael’s Hospital, 30 Bond St., Toronto, ON M5B 1W8 Canada; 2 MAP Centre for Urban Health Solutions, St. Michael’s Hospital, 30 Bond St., Toronto, ON M5B 1W8 Canada; 3 Department of Medicine, University of Toronto, Toronto, Canada; 4 National Healthcare Group Eye Institute, Tan Tock Seng Hospital, 11 Jalan Tan Tock Seng, Singapore, 308433 Singapore; 5 Singapore Eye Research Institute, Singapore, Singapore; 6 School of Material Science and Engineering, Nanyang Technological University, Singapore, Singapore; 7 Department of Global Health, University of Washington, 325 Ninth Ave, Seattle, WA 98104 USA; 8 Division of Allergy and Infectious Diseases, University of Washington, 325 Ninth Ave, Seattle, WA 98104 USA; 9 Division of Rheumatology, Columbia University, Vagelos College of Physicians & Surgeons, 630 W 168th St, P&S 3-450, New York, NY USA; 10 Bill & Melinda Gates Foundation, PO Box 23350, Seattle, WA 98102 USA

## Abstract

An unprecedented volume of research has been generated in response to the COVID-19 pandemic. However, there are risks of inefficient duplication and of important work being impeded if efforts are not synchronized. Excessive reliance on observational studies, which can be more rapidly conducted but are inevitably subject to measured and unmeasured confounders, can foil efforts to conduct rigorous randomized trials. These challenges are illustrated by recent global efforts to conduct clinical trials of post-exposure prophylaxis (PEP) as a strategy for preventing COVID-19. Innovative strategies are needed to help overcome these issues, including increasing communication between the Data Safety and Monitoring Committees (DSMCs) of similar trials. It is important to reinforce the primacy of high-quality trials in generating unbiased answers to pressing prevention and treatment questions about COVID-19.

The proliferation of research on COVID-19 treatment and prevention since SARS-CoV-2 first emerged has been unprecedented. Since SARS-CoV-2 first emerged in Wuhan, China, and triggered a global pandemic, nearly 3000 trials have been registered on ClinicalTrials.gov. While this volume of research is impressive, there are risks of inefficient duplication, and important work may also be impeded if efforts are not synchronized. Ongoing efforts to conduct rigorous randomized trials of COVID-19 post-exposure prophylaxis (PEP) provide illustrative examples.

PEP is a commonly used strategy for the prevention of infectious diseases, in which people who have recently been exposed to a pathogen use a short course of targeted, antimicrobial chemotherapy to decrease the chance of acquiring infection. In the absence of a preventive vaccine against SARS-CoV-2, PEP is an intuitively attractive option to impact the progression of the epidemic, and numerous research groups around the globe have launched randomized controlled trials.

Based on pre-clinical data suggesting in vitro inhibition of both SARS-CoV and SARS-CoV-2 [1, 2], plausible mechanisms of action including the alkalinization of endosomes required for viral replication [3], and early clinical case series from France suggesting a reduction in the SARS-CoV-2 viral load in the upper respiratory tract [4], chloroquine (CQ) and hydroxychloroquine (HCQ) have been leading candidates for COVID-19 PEP. The HIV protease inhibitor lopinavir/ritonavir is also being evaluated, based on reports showing in vitro activity against SARS-CoV [5], and observational data suggesting efficacy in humans as PEP against MERS-CoV [6]. At least 16 clinical trials of HCQ/CQ COVID-19 PEP have been planned or initiated worldwide (Fig. 1).

**Fig. 1:**
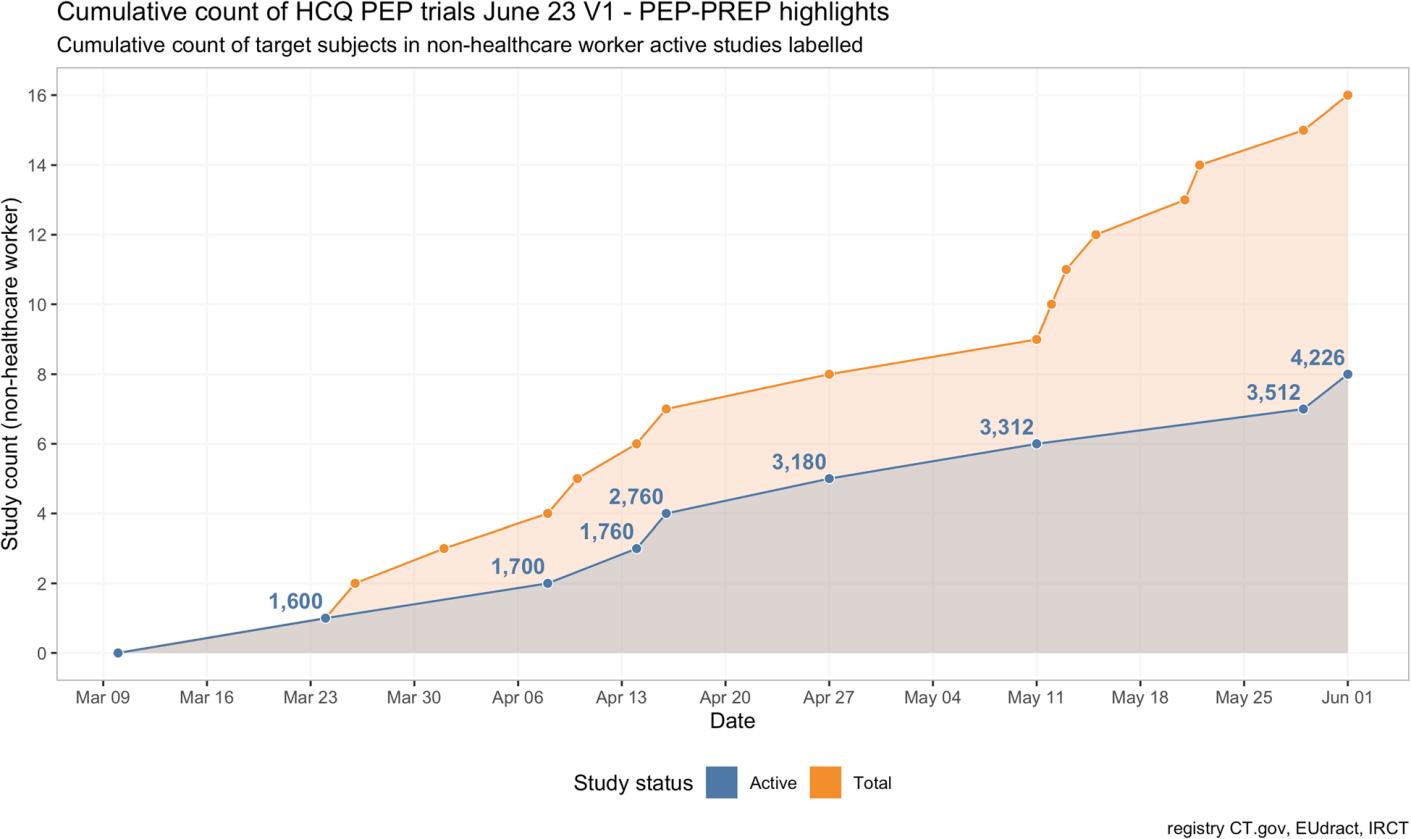
Cumulative count of HCQ PEP trials

The first of these trials has already been completed and showed no difference in the incidence of confirmed or probable COVID-19 at day 14 in participants receiving 5 days of HCQ compared to placebo [7]. An important limitation was that confirmatory testing for SARS-CoV-2 was not widely available, such that the primary outcome was confirmed by PCR in < 15% of cases. The study was underpowered to assess the impact among household members alone, among whom PEP decreased COVID-19-like illness by ~ 30% but the result was not statistically significant. Intriguingly, in pre-defined subgroup analyses, PEP efficacy appeared greater when taken 1–2 days compared to 3–4 days after exposure, supporting biological plausibility of increased efficacy with early PEP, although results were not statistically significant. A second, cluster-randomized HCQ PEP trial in Spain showed no difference in either PCR-confirmed symptomatic COVID-19 disease or SARS-CoV-2 infection among asymptomatic contacts [8]. Limitations of this well-conducted trial include that randomization was performed prior to obtaining informed consent, such that allocation concealment may have been threatened, and the open-label nature of the intervention.

Additional data are urgently needed to resolve this important question. Unfortunately, many of the rigorously designed trials that sought to address it have been thwarted by provocative observational studies questioning the cardiac safety profile of these drugs. Most notably, a prominent publication in *The Lancet* that claimed to be based on data from 96,032 patients on six continents concluded that HCQ and CQ offered no clinical benefit, increased ventricular arrhythmias, and decreased in-hospital mortality among COVID-19 patients [9]. However, careful scrutiny revealed major flaws, and the veracity of the primary data could not be verified by the authors [10]. Though retracted within 2 weeks, the initial report garnered media headlines worldwide, and regulatory authorities halted trials in several countries.

However, the problem predates the retraction of *The Lancet* article. Indeed, several well-conducted cohort studies had also suggested a lack of clinical benefit, and/or the potential for arrhythmias with HCQ/CQ when used for COVID-19 treatment, sometimes using propensity score weighting in an attempt to minimize bias [11, 12]. These findings, related media reports, and associated changes in regulatory guidance foiled other efforts to conduct rigorous COVID-19 PEP trials, including one in Asia and another in New York City. This phenomenon should be a cause for alarm. Some degree of measured and unmeasured confounding may always be present in non-randomized studies—a problem that only careful randomization and appropriate allocation concealment can adequately address. Observational studies may raise important hypotheses and may lead to the introduction of additional safeguards in trials [13], but should not impede their continuation.

Innovative solutions have been proposed to address these epistemological challenges, including increasing communication between the Data Safety and Monitoring Committees (DSMCs) of similar trials. DSMCs are charged with advising on the net ratio of benefits to risks of ongoing trials, yet are constrained by their access to limited data from a single trial. While meta-analysis can be used to pool results from multiple studies retrospectively, a unique opportunity to detect emerging safety and efficacy signals in real-time arises when trials are conducted simultaneously. The World Health Organization thus convened a series of consultations about COVID-19 drug prevention trials in early 2020 [14]. In addition to harmonizing study procedures and endpoints, a key objective was to build a “Core DSMC” that could oversee multiple trials and/or facilitate the confidential sharing of DSMC reports between trials. The rapid sharing of preliminary DSMC reports from several HCQ chemoprophylaxis trials was instrumental in moving past the initial roadblock posed by *The Lancet* publication cited above.

During a global pandemic, the need for scientific rigor is particularly acute. The complexity and longer timelines associated with conducting randomized trials create a strong incentive to rely on observational analyses for clinical guidance, which are easier and quicker to complete. Nevertheless, we must vocally reinforce the primacy of high-quality trials in generating unbiased answers to pressing prevention and treatment questions about COVID-19.

## Data Availability

Not applicable
